# ASO Author Reflections: Can We Preserve Fertility without Compromising Oncologic Outcomes?: Preoperative Oncofertility for ER-Positive Breast Cancer

**DOI:** 10.1245/s10434-026-20373-1

**Published:** 2026-08-04

**Authors:** Jennifer H. Chen, Irene Peregrin-Alvarez, Carla L. Warneke, Laurie J. McKenzie, Terri L. Woodard, Helen M. Johnson

**Affiliations:** 1 Department of Breast Surgical Oncology, The University of Texas MD Anderson Cancer Center, 1400 Pressler Street, FCT7.5000, Houston, TX 77030 USA; 2 Department of Gynecologic Oncology and Reproductive Medicine, The University of Texas MD Anderson Cancer Center, Houston, TX USA; 3 Department of Obstetrics and Gynecology, Baylor College of Medicine, Houston, TX USA; 4 Department of Biostatistics, The University of Texas MD Anderson Cancer Center, Houston, TX USA

## Past

Many women of childbearing age diagnosed with breast cancer face the challenge of balancing timely cancer treatment with desire to preserve future fertility.^1^ Current guidelines recommend oncofertility counseling prior to initiation of systemic cytotoxic therapies.^2–4^ However, incorporation of these recommendations into actual clinical practice remains variable. For patients with breast cancer, data supporting the oncologic safety of fertility preservation (FP) in the neoadjuvant setting are particularly scarce, and fear of disease progression and/or delay in initiation of cancer treatment serve as major barriers to pursuing FP among both patients and providers.^5,6^ Given the known mitogenic impact of estrogen in breast cancer, there remains concern that high supraphysiologic levels of estrogen during FP may inadvertently promote tumor growth and dissemination with the primary tumor in situ.

## Present

The goal of this study was to evaluate the oncologic safety of performing FP among women with in situ estrogen receptor (ER)-positive breast cancer.^7^ We performed two analyses comparing (1) the timing of FP (preoperative versus postoperative; i.e., with tumor in situ versus resected) and (2) receipt of neoadjuvant systemic therapy with or without FP. Our data demonstrated that timing of FP did not significantly impact 5-year progression-free survival and disease-specific survival. Importantly, no events of disease progression occurred in the neoadjuvant period after FP receipt but before tumor resection. While patients undergoing FP experienced a 2-week delay in neoadjuvant treatment receipt, this modest delay did not translate into a significant difference in survival outcomes.

## Future

These results support the oncologic safety of preoperative FP in a dedicated cohort of patients with ER-positive breast cancer and hold important implications for oncofertility counseling. Future efforts to corroborate these findings should include larger study cohorts with longer-term follow-up to ensure sustained oncologic safety. Barriers to FP use—including access^8^ and patient and physician perceptions of FP risk^5^—also warrant further investigation. As the incidence of breast cancer in young adults rise,^9,10^ demand for fertility preservation is expected to increase in the coming years.^11^ Therefore, we as a surgical oncology community have an obligation to mitigate the burden of infertility for our current and future patients.
